# Apolipoprotein E polymorphisms and primary glaucoma in Saudis

**Published:** 2009-05-04

**Authors:** Najwa Mohammed Al-Dabbagh, Nourah Al-Dohayan, Misbahul Arfin, Mohammad Tariq

**Affiliations:** 1 Department of Ophthalmology, Armed Forces Hospital, Riyadh, Saudi Arabia; 2 Research Center, Armed Forces Hospital, Riyadh, Saudi Arabia

## Abstract

**Purpose:**

The frequencies of apolipoprotein E (*APOE*) alleles and genotypes were examined in 230 Saudi subjects including primary open-angle glaucoma (POAG; n=60) and primary angle-closure glaucoma (PACG; n=40) patients as well as 130 control subjects.

**Methods:**

The presence of glaucoma in patients was based on clinical examination and/or ophthalmic records. The *APOE* allele frequency (ε2, ε3, and ε4) was studied by polymerase chain reaction (PCR) followed by reverse-hybridization and restriction fragment length polymorphism techniques.

**Results:**

Analysis of data showed a complete absence of ε2 allele and a significantly lower frequency of the ε3 allele in primary glaucoma patients (90.5%) compared to the control subjects (95.7%, p=0.034, relative risk [RR]=0.473, protective fraction [PF]=0.318). The frequency of the ε4 allele was significantly higher in the glaucoma patients (9.5%) compared to the control subjects (4.2%, p=0.034, RR=2.169, etiological fraction [EF]=0.329). The ε3/ε3 genotype was more common in controls than patients (p=0.060, RR=0.465, PF=0.322). The difference in genotype (ε3/ε4) was not statistically significant between the two groups (p=0.283). Genotype ε4/ε4 was found only in 3% of patients while being completely absent in the controls (p=0.080). The genotypes, ε2/ε2, ε2/ε3, and ε2/ε4, were absent in both the test and control groups. When patients were divided on the basis of types of glaucoma, POAG patients had a significantly higher frequency of ε4 allele and ε4/ε4 genotype than controls whereas there was no significant difference between PACG patient and control groups in frequencies of *APOE* alleles and genotypes.

**Conclusions:**

This study indicates that the ε4 allele may be associated with POAG and could be a risk factor while ε3 may be protective for POAG, and APOE polymorphisms may not be associated at all with PACG in Saudis.

## Introduction

Primary glaucoma (PG) is one of the most common eye diseases that may potentially result in bilateral blindness. It has been estimated that nearly 66.8 million people worldwide (around 2%) are affected by glaucoma [1,2]. The disease is characterized by optic nerve atrophy with an excavated optic nerve head and progressive visual field loss. The loss of retinal ganglion cells is the typical pathology in glaucoma [3,4]. Although elevation of intraocular pressure (IOP) is often related to the optic nerve damage in glaucoma [5], factors other than IOP are likely to have a role in the pathogenesis of glaucomatous optic neuropathy, particularly in individuals with normal tension glaucoma (NTG). Evidence from population and family studies supports heredity of glaucoma to be a complex trait. It is a genetically heterogeneous disorder attributed to the effects of individual causative mutations as well as interactions of multiple genes with a variety of environmental factors [6].

Apolipoprotein E (APOE) is the major apolipoprotein in the central nervous system, which plays an important role in the uptake and redistribution of cholesterol within neuronal network [7]. APOE is synthesized by Müller cells (the predominant glial cells of the retina), released into the vitreous, and then transported into the optic nerve through anterograde rapid transport where it has an important role in axonal nutrition [8]. It has been suggested that APOE plays a role in neuronal survival following ischemia and other chemical insults and that a particular APOE isoform may be related to neuronal degeneration in glaucoma [9]. The gene encoding APOE has three polymorphic variants in humans, these variants are designated as ε2, ε3, and ε4. These variants differ from one another by the presence of either a C or T nucleotide at codons 112 and 158. These three alleles encode different APOE isoforms, which vary significantly in structure and function including receptor binding capacity and lipid metabolism [10]. As each individual human being carries two allelic copies in a gene, six possible genotypes (ε2/ε2, ε3/ε3, ε2/ε3, ε3/ε4, ε2/ε4, and ε4/ε4) are formed by different combinations of these three alleles. The frequency of these genotypes differ significantly among different ethnic groups. However, APOE ε3/ε3 is the most predominant genotype and e3 the most common allele in majority of populations [11,12].

Earlier studies clearly point toward a possible association between *APOE* alleles and glaucoma. However, the results of these studies are contradictory. Some investigators show positive association [9,13,14] while others show no link at all [15-17]. More over, earlier studies were mainly restricted to White populations from Australia [9], United Kingdom [15,16], and Sweden [17] with only few reports from other ethnic groups [13,14]. So far, no attempt has been made to study a possible association between *APOE* alleles and glaucoma in a Saudi population.

## Methods

### Subjects

The present study was undertaken to evaluate the association of *APOE* alleles and genotype with glaucoma in Saudi primary glaucoma patients. A total of 100 unrelated Saudi patients with primary glaucoma (primary open-angle glaucoma [POAG] and primary angle-closure glaucoma [PACG]) were recruited from the Ophthalmology Clinic of the Riyadh Military Hospital (Riyadh, Saudi Arabia). The patient group consisted of 47 males and 53 females with the age at diagnosis ranging from 30 to 78 years (mean±SD: 58±14.4 years). The control group consisted of 130 unrelated subjects with 102 males and 28 females and their ages ranging from 20 to 58 years (mean±SD: 45±11.6 years). The diagnosis of PG was based on clinical observations**.**

A comprehensive eye examination was done that included best-corrected visual acuity (BCVA) measurements using the logarithm of the minimum angle of resolution (logMAR) 4-m charts (Light House Low Vision Products, New York, NY), applanation tonometry, gonioscopy, dilated fundus examination, optic disc photography, and visual field (VF) examination. On gonioscopy, an angle was considered occludable if the pigmented trabecular meshwork was not visible at an angle greater than 180° in dim illumination. Laser iridotomy was performed in subjects with occludable angles after consent was obtained. The ocular biometric measurements were carried out on the following day.

### Visual fields

Automated VFs were performed for all subjects with a BCVA of 4/16 (logMAR 0.6) or better using frequency-doubling perimetry (Carl Zeiss Meditec Inc., Dublin, CA). All eligible subjects underwent C-20-1 screening (if the results were unreliable or abnormal, the test was repeated) and the N-30 threshold test. The reliability criteria were no fixation or false-positive errors for the C-20-1 screening test and less than 20% fixation errors and less than 33% false-positive and false-negative errors for the threshold N-30 test. Visual fields with no depressed points to any level of sensitivity were considered to be normal. A provisional diagnosis of suspected glaucoma was made when the subject had one or more of the following conditions: intraocular pressure (IOP) ≥21 mmHg in either eye, vertical cup-to-disc ratio (VCDR) ≥0.7 in either eye or cup-to disc ratio (CDR) asymmetry ≥0.2, and focal thinning, notching, or a splinter hemorrhage. All these subjects were asked to perform a threshold VF test using the Swedish interactive threshold algorithm Standard 30-2 program (model 750, Carl Zeiss Meditec Inc.). A glaucomatous field defect was diagnosed using a single reliable threshold VF examination of the central 30° (Swedish interactive threshold algorithm Standard 30-2). The field was considered to be abnormal if the Glaucoma Hemifield test results were outside normal limits and if  three or more abnormal contiguous non-edge points (except the nasal horizontal meridian) were depressed to p<0.05 [18]. Reliability criteria were as recommended by the instrument’s algorithm (fixation losses <20%; false-positive and false-negative <33%).

### Diagnostic definitions

The distribution of VCDR and IOP was obtained from those subjects with reliable and normal supra-threshold VF testing using frequency-doubling perimetry. Cases of glaucoma were defined using the International Society of Geographical and Epidemiologic Ophthalmology classification [19]. Glaucoma was classified according to three levels of evidence. In category 1, diagnosis was based on structural and functional evidence. Using the Swedish interactive threshold algorithm 30-2, it required CDR or CDR asymmetry greater than 97.5th percentile for the normal population, or a neuroretinal rim width reduced to 0.1 CDR (between 10- and 1-o’clock or 5- and 7-o’clock) with a definite VF defect consistent with glaucoma. Category 2 was based on advanced structural damage with unproven field loss. This included those subjects in whom VFs could not be determined or were unreliable, and subjects  with CDR or CDR asymmetry greater than 99.5th percentile for the normal population. Lastly, category 3 consisted of persons whose optic discs could not be examined because of media opacities and with an IOP greater than 99.5th percentile for the normal population.

Blindness was defined as a best-corrected logMAR visual acuity of less than 2/40 (log MAR 1.3) and/or constriction of the VF to less than 10° from fixation in the better eye [20]. Hyperopia was defined as spherical equivalent greater than 0.50 diopters in a phakic eye [21]. Diabetes mellitus was detected based on the current use of antidiabetic medication and/or a random blood glucose level greater than 200 mg/dl [22].

Thus, the primary glaucoma patients were separated into two groups (POAG and PACG) using the following criteria. POAG: The patients with open anterior chamber angles on gonioscopy, typical glaucomatous cupping of optic disc and visual field defects characteristic of glaucoma were classified as POAG. PACG: The patients with partially or totally closed anterior chamber angle (appositional angle closure or synechiae in angle) along with damaged glaucomatous optic disc or defective glaucomatous visual field were classified as PACG. Subjects with any sign of secondary angle closure (e.g., uveitis, lens related glaucoma; microspherophakia; evidence of neovascularization in the angle and associated retinal ischemia or congenital angle anomalies) were excluded. Patients with signs of intracranial disease that would cause optic nerve atrophy in X-ray computerized tomography or magnetic resonance imaging were also excluded.

Venous blood was collected and stored at −20 °C before the extraction of DNA. The study protocol was approved by the ethics committee of Riyadh Military Hospital, and informed consent was obtained from all study participants.

### Genotyping

The genotypes of the *APOE* polymorphisms were determined using the ApoE StripAssay^TM ^kit (ViennaLab Labordiagnostika GmbH, Vienna, Austria), which is based on polymerase chain reaction (PCR) and reverse-hybridization techniques. The procedure included three steps: (1) DNA isolation, (2) PCR amplification using biotinylated primers, and (3) hybridization of amplification product to a test strip containing allele-specific oligonucleotide probes immobilized as an array of parallel lines. Bound biotinylated sequences were detected using streptavidin-alkaline phosphatase and color substrates. To cross-check the results, the genotypes of the *APOE* polymorphisms were also determined by PCR and restriction fragment length polymorphism (RFLP) technique. Primers were designed on the basis of the sequence data for *APOE* available in GenBank to amplify the coding sequence of *APOE*. PCR was performed using PuRe Taq Ready-To-Go PCR Beads (Amersham Biosciences, Piscataway, NJ) with the following primers: forward primer: 5- GAC GCG GGC ACG GCT GTC CAA GGA GCT GCA GGC GAC GCA GGC CCG GCT GGA CGC GGA CAT GGA GGA-3 and reverse primer: 5 - AGG CCA CGC TCG ACG CCC TCG CGG GCC CCG GCC TGG TAC ACT-3.

Genomic DNA was extracted from whole blood using a commercial kit (QIAmp; Qiagen, Hilden, Germany). Genomic DNA (200-300 ng) was used as a template in 25 μl reactions. Genomic DNA was amplified for 40 cycles. Each cycle consisted of 94 °C for 30 s, 68 °C for 10 s, and 72 °C for 1 min. PCR products obtained were separated by electrophoresis on 1.5% agarose gel in Tris-acetic acid-EDTA (TAE) buffer and visualized by ethidium bromide fluorescence. Fragments with the expected size were cut from the gel and purified using GFX PCR DNA Gel band purification kit (Amersham). Purified DNA was digested with the CfoI (HhaI) enzyme and separated by agarose gel electrophoresis to identify the genotype. On the basis of size and number of various fragments generated, *APOE* genotypes were determined as ε2/ε2 with 144 bp and 96 bp fragments; ε3/ε3 with 144 bp and 48 bp fragments; ε4/ ε4 with 72 bp and 48 bp fragments; ε2/ε3 with 144 bp, 96 bp, and 48 bp fragments; ε3/ ε4 with 144 bp, 72 bp, and 48 bp fragments; and ε2/ε4 with 144 bp, 96 bp, 72 bp, and 48 bp fragments. The prevalence of various genotypes in patients and controls was determined. Complete matching of results was obtained following both of the above mentioned procedures.

### Statistical analysis

Frequencies of various alleles and genotypes for each polymorphism were compared between patients and controls and then analyzed by Fisher’s exact test (p values less than 0.05 were considered significant). The strength of the association of disease with respect to a particular antigen is expressed by an odds ratio interpreted as relative risk (RR) according to Woolf's method as outlined by Schallreuter et al. [23]. The RR was calculated only for those alleles and genotype that were increased or decreased in glaucoma patients as compared to normal Saudis.

### Etiologic fraction

The etiologic fraction (EF) indicates the hypothetical genetic component of the disease. Values greater than 0.0 were considered significant. It was calculated for positive association (RR>1) [24].

### Preventive fraction

The preventive fraction (PF) indicates the hypothetical protective effect of one specific antigen for the disease. It was calculated for negative association (RR<1) [24]. Values less than 1.0 indicated the protective effect of the genotype/allele against the manifestation of the disease.

## Results

Out of 100 PG patients, 60 were diagnosed as having POAG and 40 as having PACG. Diagnosis of POAG was based on category 1 in nine subjects (15%) and category 2 in 51 subjects (85%). Between category 1 and category 2, there were no significant differences in age, IOP, or gender distribution. One subject was blind in both eyes, and one subject had unilateral blindness due to POAG. There were 40 subjects with PACG. Diagnosis was based on category 1 in 10 subjects (25%), category 2 in 28 subjects (70%), and category 3 in two subjects (5%). Two subjects (5%) were bilaterally blind and three (7.5%) were unilaterally blind, all due to PACG.

The frequency results of *APOE* alleles and genotypes in the PG patients and the control subjects are summarized in Table 1 through Table 6. The frequency of the ε3 allele was significantly lower in the glaucoma patients (90%) than in the control subjects (95.7%, p=0.034, RR=0.473, PF=0.318). In contrast, the frequencies of the ε4 allele was significantly higher in the glaucoma patients than in the controls (9.5% versus 4.2%, p=0.034, RR=2.169, EF=0.329). The allele ε2 was absent in both the patient and control groups (Table 1).

**Table 1:** Distribution of APOE allele frequencies in glaucoma patients and matched controls.

**Allele**	**Glaucoma (n=200)**		**Control (n=260)**		**p value**	**RR**	EF*/PF
	**Number**	**Frequency (%)**	**Number**	**Frequency (%)**			
**ε4**	19	9.5	11	4.2	0.034	2.169	0.329*
**ε3**	181	90.5	249	95.7	0.034	0.473	0.318
**ε2**	0	0	0	0	**-**	-	-

Number of subjects=n. Values with  asterisk (last column) correspond to etiological fraction (EF) while values without asterisk indicate  protective fraction (PF).

**Table 6:** Distribution of *APOE* genotype and allele frequencies in patients with PACG and matched controls.

**Genotype/Allele**	**Angle closure glaucoma (n=40)**	**Controls (n=130)**	**p value**	**RR**	EF*/PF
**ε3/ε4**	6 (15)	11 (8.4)	0.236	1.909	0.167*
**ε4/ε4**	0	00	-	-	-
**ε3/ε3**	34 (85)	119 (91.5)	0.236	0.523	0.168
**ε4**	6 (7.5)	11 (4.2)	0.246	1.835	0.160*
**ε3**	74 (92.5)	249 (95.7)	0.246	0.544	0.161

Number of subjects=n; values are indicated as n (%). Values with  asterisk (last column) correspond to etiological fraction (EF) while values without asterisk indicate  protective fraction (PF).

Our study on various genotypes of *APOE* also showed variations in patient and control groups (Table 2). The prevalence of ε3/ε3, ε3/ε4, and ε4/ε4 was 84%, 13%, and 3% in patients and 91.5%, 8.4%, and 0% in the control group, respectively. Though the ε3/ε3 genotype was more common in both the test and control Saudi population, the statistical analysis of data showed a nearly significant difference in ε3/ε3 genotype frequencies between patients and controls (p=0.060, RR=0.465, PF=0.322). The difference in the frequencies of the second common genotype (ε3/ε4) was not statistically significant between the two groups (p=0.283). Genotype ε4/ε4 was found only in 3% of patients while being completely absent in the controls (p=0.080). The genotypes ε2/ε2, ε2/ε3, and ε2/ε4 were absent in both the groups. These results indicated that the ε4 allele is associated with glaucoma and can be a risk factor while the ε3 allele may be protective in Saudis. The frequencies of various genotypes and alleles were almost similar in male and female patients, clearly indicating that gender plays no role in genotype/allele distributions among populations (Table 3).

**Table 2:** Distribution of *APOE* genotype frequencies in glaucoma patients and matched controls.

**Genotype**	**Glaucoma (n=100)**		**Control (n=130)**		**p value**	**RR**	EF*/PF
	**Number**	**Frequency (%)**	**Number**	**Frequency (%)**			
**ε3/ε3**	84	84	119	91.5	0.06	0.465	0.322
**ε3/ε4**	13	13	11	8.4	0.283	1.616	0.2063*
**ε4/ε4**	3	3	0	0	0.08	-	-
**ε2/ε2**	0	0	0	0	-	-	-
**ε2/ε3**	0	0	0	0	-	-	-
**ε2/ε4**	0	0	0	0	-	-	-

Number of subjects=n. Values with  asterisk (last column) correspond to etiological fraction (EF) while values without asterisk indicate  protective fraction (PF).

**Table 3:** Comparison of *APOE* genotypes and alleles frequencies in male and female glaucoma patients.

**Genotype/Allele**	**Male (n=48)**		**Female (n=52)**		**p value**
	**Number**	**Frequency (%)**	**Number**	**Frequency (%)**	
**ε3/ε3**	40	83.33	44	84.61	0.999
**ε3/ε4**	8	16.66	5	9.61	0.377
**ε4/ε4**	0	0	3	5.76	0.243
**ε3**	88	91.66	93	89.42	0.636
**ε4**	8	8.33	11	10.57	0.636

Number of subjects=n.

Though the distribution of *APOE* genotypes and alleles was not significantly different in the two types of glaucoma (Table 4), significant difference was found in the frequencies of the ε4/ε4 genotype and the ε4 and ε3 alleles between the POAG patients and controls when each glaucoma group was compared with the controls separately. The frequencies of the ε4/ε4 genotype and the ε4 allele were significantly higher in POAG patients than in controls (p=0.03 and p=0.02, respectively; Table 5). The frequency of the ε3 allele was significantly higher in controls (p=0.02). Moreover, the frequencies of various *APOE* genotypes and alleles differed between PACG and controls, but the differences were not statistically significant (Table 6), indicating that the ε4/ε4 genotype and ε4 allele are only significantly associated with POAG and not with PACG in Saudis.

**Table 4:** Comparison of *APOE* genotype and allele frequencies in patients with POAG  and PACG.

**Genotype/Allele**	**Open angle glaucoma (n=60)**	**Angle closure glaucoma (n=40)**	**p value**
**ε3/ε4**	7 (11.66)	6 (15)	0.76
**ε4/ε4**	3 (5.00)	0	0.27
**ε3/ε3**	50 (83.33)	34 (85)	0.99
**ε4**	13 (10.83)	6 (7.5)	0.47
**ε3**	107 (89.16)	74 (92.5)	0.47

Number of subjects=n; values are indicated as n (%).

**Table 5:** Distribution of *APOE* genotype and allele frequencies in patients with POAG and matched controls.

**Genotype/Allele**	**Open angle glaucoma (n=60)**	**Controls (n=130)**	**p value**	**RR**	EF*/PF
**ε3/ε4**	7 (11.66)	11 (8.4)	0.59	1.428	0.113*
**ε4/ε4**	3 (5.00)	00	0.03	-	-
**ε3/ε3**	50 (83.33)	119 (91.5)	0.13	0.462	0.255
**ε4**	13 (10.83)	11 (4.2)	0.02	2.75	0.034*
**ε3**	107 (89.16)	249 (95.7)	0.02	0.363	0.034

Number of subjects=n; values are indicated as n (%). Values with  asterisk (last column) correspond to etiological fraction (EF) while values without asterisk indicate  protective fraction (PF).

## Discussion

The results of this study showed the complete absence of the *APOE* ε2 allele but a very high frequency (95.7%) of ε3. The frequency of ε4 (known as the thrifty allele) was 4.3% in this Saudi population. Global studies on the *APOE* locus have shown highly significant variations in the allele frequencies of ε2 (0%–12%), ε3 (75%–90%), and ε4 (6%–20%) [25-31]. The reason for the dissimilar findings in different populations is far from clear and may reflect a regional difference in the *APOE* genotype frequencies. The ε3 allele is the most frequent in all the human groups, especially in populations with a long established agricultural economy whereas the *APOE* ε4 allele remains higher in populations where the economy of foraging still exists or where the food supply is/was scarce and sporadically available [32].

Results of the present study revealed significant differences in the frequencies of ε3 and ε4 alleles between the glaucoma patient and the control group (Table 1). The ε3 allele was more common in controls while the ε4 allele was predominant in glaucoma patients, suggesting that the inheritance of the ε4 allele might be a risk factor whereas ε3 may exert a protective effect for glaucoma in the Saudi population. Our findings are in agreement with Yilmaz et al. [33] who reported a protective role of APOE ε3 allele in patients with exfoliation syndrome (a disease closely associated with glaucoma) in the Turkish population. The neuroprotective effect of ε3 is evident from several earlier studies [34,35]. APOE has an isoform-specific effect on neuronal growth with ε3 stimulating neuronal elongation and neurite outgrowth on the dorsal root ganglion [34]. In individuals with acute cerebral ischemia such as an intracerebral hemorrhage, the ε3 allele confers a much higher rate of survival and functional recovery whereas ε4 leads to a higher rate of disability and mortality [35].

Moreover, the results of this study clearly suggest that the presence of ε4 has a negative impact as its presence was found to be associated with high risk of POAG. Vickers et al. [9] also reported an association between the ε4 allele and NTG in the Tasmanian population. Recently, Yaun et al. [36] reported that the ε4 allele may be a latent risk factor in developing primary glaucoma in the Chinese population. On the other hand, Liew et al. [37] found a weak association between *APOE* ε4 and retinal microvascular degeneration.

Contrary to these findings, a decreased risk of NTG in Chinese [13,14] and POAG in Japanese [38] has been reported whereas some investigators reported no link between *APOE* polymorphisms and glaucoma. Besides glaucoma, the *APOE* ε4 allele has been identified as a genetic susceptibility factor for a variety of neurodegenerative disorders in diverse ethnic populations [39-42]. The *APOE* ε4 allele has also been associated with early age-at-onset of Alzheimer’s Disease (AD) in a dose-dependent manner [43,44]. Interestingly, a high incidence of glaucoma in AD patients clearly suggests a close association between these neurodegenerative disorders [45,46]. It has been hypothesized that the cellular mechanisms involved in the degeneration of optic nerve cells in glaucoma are quite similar to the neurodegenerative changes in AD [9,47,48]. The *APOE* ε4 allele is also strongly linked with increased risk of Parkinson disease, schizophrenia, and coronary artery disease [49-55]. Possession of the ε4 allele is also associated with a retarded recovery after traumatic head injury [56,57].

*APOE* alleles modulate the biological functions of APOE in part by altering the binding of the different lipoprotein lipid classes [49]. Individuals carrying the ε4 allele have higher plasma and neuronal levels of cholesterol as compared to individuals with the ε2 or ε3 allele. The higher frequency of the ε3/ε3 genotype in controls when compared to the glaucoma patients indicated a protective effect of ε3/ε3 against the development of glaucoma in Saudis. On the other hand, the ε4/ε4 genotype was only found in the patient group with a complete absence in the normal Saudi population, suggesting its association with POAG. The genotypes ε2/ε2, ε2/ε3, and ε2/ε4 were absent in both the patient and control groups. Earlier studies on *APOE* polymorphisms in the general population also showed the absence of genotypes containing the ε2 allele among Saudis [58] as well as Native Americans [59].

The results of this study suggest that *APOE* alleles may influence the risk of POAG but has no effect on the susceptibility of PACG. The inheritance of the ε4 allele is associated with elevated risk of POAG but not of PACG, and ε3 may exert protection against POAG. However, further studies involving larger number of patients are warranted to reach a definite conclusion.
